# Temporomandibular disorders in patients with HIV: a cross-sectional study

**DOI:** 10.1016/j.bjid.2024.103769

**Published:** 2024-06-08

**Authors:** Monah Sampaio Santos, Larissa Souza Santos-Lins, Sávio Vinicius Burity Amorim Nunes Amaral, Carlos Brites, Liliane Lins-Kusterer

**Affiliations:** Faculdade de Medicina da Universidade Federal da Bahia (FMB-UFBA), Programa de Pós-Graduação em Medicina e Saúde, Salvador, BA, Brazil

**Keywords:** Temporomandibular disorder, HIV, Depression

## Abstract

Untreated HIV infection leads to severe immunodeficiency and can be associated with an accelerated aging process and a higher prevalence of frailty. Systemic changes are known to cause greater oral manifestations and decreased orofacial function. However, there is no investigation on Temporomandibular Disorders (TMD) in this population. This study aims to assess the prevalence of TMD in individuals living with HIV/AIDS. This cross-sectional study included HIV patients, with undetectable plasma viral load, under follow-up in the infectious disease's outpatient clinic at the Federal University of Bahia hospital. We recorded socio-demographic data, Fried's frailty criteria, Research Diagnostic Criteria for Temporomandibular Disorder, and Beck's Depression Inventory (BDI) through the application of structured questionnaires and extra-oral examination findings. Data analysis was conducted on SPSS-v18. The sample consisted of 198 patients. The prevalence of TMD was (33.8 %), most affecting females (46.6 %). Difficulty in opening the mouth and parafunctional habits were the main symptoms of the disease, as well as functional limitations. The mean of the BDI score was higher in TMD group than in those without TMD (11.01 ± 8.61 vs. 7.60 ± 7.52 valor de *p* = 0.004). Logistic regression showed an association between sex (OR=2.305, 95 % CI 1.243‒4.275) and depression (OR = 1.045, 95 % CI 1.005‒1.087) and TMD in HIV patients. The present study observed the prevalence of symptoms associated with TMD as difficulty opening the mouth, muscle fatigue, and joint noises in patients with chronic HIV and associated with depression. Highlights the importance of a broader view of the health of individuals living with HIV.

## Introduction

Temporomandibular Disorder (TMD) is a group of diseases of the stomatognathic system involving the temporomandibular joint, muscles, and attached structures such as disc and ligaments, which may be or not be associated with biological factors, psychological factors, and/or systemic morbidities.^1^^,^^2^ The prevalence of TMD is estimated at 31 % in the general population and plays a important role in affected individuals.^3^ Pain, limitation, and loss of function are findings that can interfere in the quality of life of these patients.^4^^,^^5^ Disease mechanisms are diverse, and some studies have linked TMD to depression.^6^^,^^7^

Human Immunodeficiency Virus (HIV) characterized by a progressive loss of CD4+ *T*-cells, that makes the host progressively susceptible to opportunistic infections and neoplasms. The advance in Antiretroviral Therapies (ART) changed the initially fatal disease in a chronic condition increasing patient's survival and quality of life.^8^

Immunological changes in HIV patients can increase the risk of more oral manifestations than those found in healthy individuals.^9^^,^^10^ The relationship between TMD and HIV has been studied by some authors, but it remains controversial.^11^, ^12^, ^13^ Metabolic modifications on muscle and bone, muscle dysfunction, and myofascial limitation are report in the literature.^12^^,^^14^^,^^15^ Some researchers detected an association between pain, masticatory muscles, and joint function in HIV.^10^^,^^16^

Regarding HIV therapies, the duration of ART was correlated with bruxism and the presence of TMD.^11^ A previous report described the development of TMD with the use of a protease inhibitor and also observed cases in which rheumatoid disease was associated with ART.^17^ In addition, a case report described the onset of joint pain after starting ART.^13^

Due to a lack of studies addressing this topic, the objective of this study is to evaluate the prevalence of TMD in HIV patients and to describe its clinical characteristics.

## Materials and methods

### Study design

This was a cross-sectional study conducted between the years 2019 to 2020.

Patients and settings: We included HIV patients followed up at the infectious diseases outpatient clinic at a university hospital, Federal University of Bahia. All included individuals agreed to participate and signed a written informed consent form.

### Inclusion criteria

To be eligible, patients must full-filled the following criteria Confirmed HIV infection, stable therapy with undetectable HIV-1 plasma viremia (< 50 copies/mL).

Exclusion criteria included individuals younger than 18 years, patients with total or partial walking limitation, unable to understand questions.

### Main study variables

Socio-demographic information was collected by using a structured questionnaire with information about age, gender, race, marital status, income and education level. Lifestyle and clinical characteristics were also recorded and included questions on smoking and alcohol consumption, clinical illness, and medication use.

### Frailty criteria

The presence of frailty was assessed by Fried's Frailty Criteria (FFC) using the parameters: Weight loss; Exhaustion; Physical activity; Muscle weakness and gait speed.^18^ Participants presenting three or more positive criteria were classified as a Frail Syndrome (FS), one or two positive criteria as a pre-frail syndrome, and participants with no positive criteria as robust or non‐frail.

### Temporomandibular dysfunction criteria

To evaluate TMD we used the Research Diagnostic Criteria for Temporomandibular Disorder (RDC/TMD), a validated instrument for TMD diagnosis in epidemiological studies.^19^ The diagnosis of TMD was based on axis I of the RDC/TMD questionnaire, which consists of a physical examination and questions about the history of orofacial pain. Subsequently, the results were analyzed from a diagnostic flowchart, for analysis purposes we classified patients in two groups: with TMD or without TMD. Data from the symptom's questionnaire present in the RDC/TMD axis II were also used to correlate the diagnosis with the presence of orofacial symptoms. The functional limitation scale, also present in axis II, was used to assess functional limitation. The score ranges from 0 to 10, with 0 being no limitation and 10 being severe limitation. For statistical analysis purpose, we considered scores between 0‒1 as the absence of limitation and from 2 to 10 presence of functional limitation.

### Depression criteria

To assess the severity of depression, the Beck Depression Inventory-II (BDI) was used. This instrument is a Likert-type scale composed of 21 items with 4 options of answer in each, resulting in a final score that classifies patients as no depression, mild depression, moderate depression, and severe depression.^20^ For analysis purposes, we classified as depressive individuals with ≥ 14 points.

### Statistical analysis

The data were stored and analyzed using the Statistical Package for the Social Sciences (SPSS) version 18. For the analysis of demographic and clinical data, descriptive statistics were used. Continuous variables were described with measures of central tendency and dispersion, expressed as means and standard deviation. Dichotomous or categorical variables were evaluated by frequency measures and expressed as percentages. The statistical significance was set in 0.05. Variables with *p* < 0.20 in the bivariate analysis were included in a model for logistic regression, using the enter method. The parameters used to assess the quality of the logistic regression were the Hosmer & Lemeshow tests (the goodness of fit) to determine the adequacy of the model (*p* > 0.5), and the Omnibus Tests (*p* < 0.05) to assess the improvement of the data in the equation with the model and NargleKerke's R2. We performed a network analysis to evaluate TMD symptoms and functional limitations associated with TMD, using the InsigSampler estimator with bivariate regression analysis, with Bootstrap 500 of the non-parametric type.

### Ethics committee

This work was approved by the Ethics Review Board, Faculty of Medicine, University of Bahia, protocol n°42,996,315.9.0000.5577.

## Results

A total of 217 patients were evaluated, of which 198 were included in the study. Six patients did not complete the questionnaires, and 3 abandoned the interview and were excluded from the sample. Table 1 summarizes the socio-demographic characteristics of included patients.

**Table 1:** Socio-demographic and clinical characteristics of 198 individuals with HIV, according to TMD status, Salvador, Brazil, 2020.

Sociodemographic and occupational characteristics	*N* = 198 (%)	With TMD (*n* = 67) (%)	Without TMD (*n* = 131) (%)	PR	p-value
Age – n (%)					
≥ 50 years	127 (64.1)	44 (34.6)	83 (65.4)	1.06	0.748
< 50 years	71 (35.9)	23 (32.4)	48 (67.6)	1	
Sex – n (%)					
Female	82 (41.4)	38 (46.6)	44 (53.7)	1.85	0.001
Male	116 (58.6)	29 (25.0)	87 (65.0)	1	
Race (194) – n (%)					
White	21 (10.8)	8 (38.1)	13 (61.9)	1.23	0.527
Pardo	92 (47.4)	33 (35.9)	59 (64.1)	1.16	0.486
Black	81 (41.8)	25 (30.9)	56 (69.1)	1	
Marital Status – n (%)					
Engaged in a stable relationship	50 (25.3)	20 (40.0)	30 (60.0)	1.26	0.288
Not engaged in a stgable relationship	148 (74.7)	47 (31.80)	101 (68.2)	1	
Income (in Brazilian Reais) (191) – n (%)					
≤ 1 MW^a^	52 (27.2)	19 (36.5)	33 (63.5)	1.08	0.724
> 1 MW^a^	139 (72.8)	47 (33.8)	92 (66.2)	1	
Education – n (%)					
High school and higher	125 (63.1)	44 (35.2)	81 (64.8)	1.02	0.886
Until elementary school	73 (36.9)	23 (31.5)	50 (68.5)	1	
Smoking – n (%)					
No	181 (91.4)	63 (34.8)	118 (65.2)	1.47	0.347
Yes	17 (8.6)	4 (23.5)	13 (76.5)	1	
Alcohol abuse – n (%)					
No	98 (49.5)	38 (38.8)	60 (61.2)	0.93	0.729
Yes	100 (50.5)	29 (29.0)	41 (71.0)	1	
Frailty Syndrome					
Yes	114 (57.6)	41 (36.0)	73 (64.0)	1.16	0.461
No	84 (42.4)	26 (31.0)	58 (69.0)	1	
Depression (BDI) – M (SD)	8.75 (8.05)	11.1 (8.61)	7.60 (7.52)	‒	0.004

TMD, Temporomandibular disorder; PR, Prevalence Ratio; MW, Minimum Wage; BDI, Beck Depression Inventory II; M, Mean; SD, Standard Deviation.

a 1USD = 5.46BR.

The prevalence of TMD in the study population was 33.8 % (*n* = 67), and it was more frequently detected in females (46.6% vs. 25 % in males, *p* = 0.001).

Some comorbidities were observed in the study's population: 4 patients had osteoarthritis, 1 fibromyalgia, 2 osteoporosis/osteopenia, and 3 thyroid diseases. All but 2 arthrosis patients also had TMD. Frailty was also observed in these patients, except for one case of arthrosis diagnosed with fibromyalgia.

No relationship was detected between smoking or alcohol abuse and TMD. In addition, frailty was diagnosed in 36 % of patients, but without difference between groups. The mean BDI score was higher in TMD group than in those without TMD (11.01 ± 8.61 vs. 7.60±7.52). Table 1 summarizes these findings.

The most prevalent symptoms related to TMD were difficulty in opening the mouth (71.4 %), parafunctional habits (67.7 %) and joint pain (66.7 %), followed by muscle fatigue (61.8 %). There was a statistical difference between the groups for these related symptoms (*p* < 0.05) with PR of 3.82 for parafunctional habits, 2.85 for joint pain, 2.67 for muscle fatigue, and 3.43 for difficulty opening the mouth (Table 2).

**Table 2:** Frequency of symptoms detected in 198 individuals with HIV, according to TMD, Salvador, Brazil, 2020.

Symptoms	*N* = 198 n (%)	With TMD (*n* = 67) n (%)	Without TMD (*n* = 131) n (%)	PR	p-value
Difficulty opening the mouth – n (%)					
Yes	21 (10.6)	15 (71.4)	6 (28.6)	2.43	>0.001
No	177 (89.4)	52 (29.4)	125 (70.6)	1	
Pain when moving the jaw – n (%)					
Yes	29 (14.6)	17 (58.6)	12 (41.4)	1.98	0.002
No	169 (85.4)	50 (29.6)	119 (70.4)	1	
Muscle fatigue – n (%)					
Yes	55 (27.8)	34 (61.8)	21 (38.2)	2.67	>0.001
No	143 (72.2)	33 (23.1)	110 (76.9)	1	
Headache – n (%)					
Yes	66 (33.3)	32 (48.5)	34 (51.5)	1.82	0.002
No	132 (66.7)	35 (26.5)	97 (73.5)	1	
Joint pain – n (%)					
Yes	48 (24.2)	32 (66.7)	16 (33.3)	2.85	>0.001
No	150 (75.8)	35 (23.3)	115 (76.7)	1	
Joint noises – n (%)					
Yes	62 (31.3)	30 (48.4)	32 (51.6)	1.77	0.003
No	136 (68.7)	37 (27.2)	99 (72.8)	1	
Parafunctional habits – n (%)					
Yes	66 (33.3)	44 (67.7)	22 (33.3)	3.82	>0.001
No	132 (66.7)	23 (17.4)	109 (82.6)	1	
Teeth fit well – n (%)					
Yes	110 (55.6)	41 (37.3)	69 (32.7)	1.26	0.254
No	88 (44.4)	26 (29.5)	62 (70.5)	1	
Feel tense or nervous – n (%)					
Yes	90 (45.5)	32 (35.6)	58 (64.4)	1.09	0.641
No	108 (54.5)	35 (32.4)	73 (67.6)	1	

TMD, Temporomandibular Disorder; PR, Prevalence Ratio.

‡ Temporomandibular joint.

The functional limitation was present in TMD, which was significantly linked to chewing solid foods (100 %, PR = 2.12, *p* ≥ 0.001), talking (80 %, PR = 2.45, *p* = 0.027), smiling (63 %, PR = 3.07, *p* = 0.004) and swallowing (60 %) (Table 3).

**Table 3:** Frequency of Functional Limitation among 198 individuals with HIV, according to TMD, Salvador, Brazil, 2020.

Functional Limitation	*N* = 198 n (%)	With TMD (*n* = 67) n (%)	Without TMD (*n* = 131) n (%)	PR	p-value
Chew solid foods – n (%)					
Yes	62 (31.3)	33 (53.2)	29 (46.8)	2.12	>0.001
No	172 (68.7)	34 (75.0)	102 (25.0)	1	
Eat soft food – n (%)					
Yes	2 (1.0)	2 (100.0)	0 (0.0)	3.01	0.046
No	196 (99.0)	65 (33.2)	131 (66.8)	1	
Open mouth to drink from a glass – n (%)					
No	198 (100.0)	67 (33.8)	131 (66.2)	‒	‒
Yes	0 (0.0)	0 (0.0)	0 (0.0)	‒	
Swallow – n (%)					
No	198 (100.0)	67 (33.8)	131 (66.2)	‒	‒
Yes	0 (0.0)	0 (0.0)	0 (0.0)	‒	
Yawn – n (%)					
Yes	5 (2.5)	3 (60.0)	2 (40.0)	1.80	0.210
No	193 (97.5)	64 (36.2)	129 (66.8)	1	
Talk – n (%)					
Yes	5 (2.5)	4 (80.0)	1 (20.0)	2.45	0.027
No	193 (97.5)	63 (32.6)	130 (67.4)	1	
Smile – n (%)					
Yes	4 (2.0)	4 (63.8)	0 (36.2)	3.07	0.004
No	194 (98.0)	63 (45.0)	131 (55.0)	1	

TMD, Temporomandibular Disorder; PR, Prevalence Ratio.

‡ Temporomandibular joint.

In the logistic regression, sex (OR = 2.305, 95 % CI 1.243‒4.275) and depression (OR = 1.045, 95 % CI 1.005‒1.087) remained significantly associated with TMD in HIV patients, as shown in Table 4. The Hosmer & Lemeshow test suggested that the model had a good fit to the data as *p* = 0.374, and the Omnibus Tests (Chi-Square = 14.91, df = 2, *p* = 0.001) showed the improvement of the equation with the model and NargleKerke's R2 suggesting that the model explains 10 % of the variance at TMD. The collinearity evaluation parameters, Tolerance index (> 0.2) and VIF (< 10) showed that the data fulfilled the prerequisites for regression with the absence of multicollinearity. Female sex (OR = 2.305) and depression (OR = 1.045) were a significantly associated with TMD.

**Table 4:** Logistic regression with TMD as the outcome related demographic and clinical characteristics among 198 individuals with HIV, Salvador, Brazil, 2020.

Variables	B	OR	95 % CI	p-value
Sex, female	0.835	2.305	(1.243‒4.275)	0.008
BDI score	0.044	1.045	(1.005‒1.087)	0.026
Constant	−1.450	0.235		< 0.001

B, Regression Coefficients; OR, Odds Ratio; CI, Confidence Interval; BDI, Beck Depression Inventory II.

Regarding signs and symptoms, logistic regression showed significant association between TMD symptoms in HIV patients and difficulty in opening the mouth (OR = 3.747, 95 % CI 1.141‒12.305), muscle fatigue (OR = 3.466, 95 % CI 1.584‒7.581) and joint noises (OR = 4.153, 95 % CI 1.793‒9.615). These symptoms were three to four times more frequent in HIV patients with TMD than without TMD (Table 5).

**Table 5:** Logistic regression with TMD as the outcome related symptoms among 198 individuals with HIV, Salvador, Brazil, 2020.

Variables	B	OR	95 % CI	p-value
Difficulty opening the mouth	1.321	3.747	(1.141‒12.305)	0.029
Pain when moving the jaw	−0.011	0.989	(0.332‒2.946)	0.984
Muscle fatigue	1.243	3.466	(1.584‒7.581)	0.002
Headache	0.380	1.462	(0.649‒3.291)	0.359
Joint pain	0.288	1.333	(0.592‒3.002)	0.487
Joint noises	1.424	4.153	(1.793‒9.615)	0.001
Parafunctional habits	−0.099	0.905	(0.404‒2.028)	0.809
Constant	−1.830	0.160		<0.001

B, Regression Coefficients; OR, Odds Ratio; CI, Confidence Interval.

## Discussion

In the present study, we detected a higher prevalence of TMD in HIV patients, with a predominance in females, and patients older than 50 years. Depression was significantly associated with TMD. The most frequent symptoms found in TMD were difficulty in opening the mouth, parafunctional habits, joint pain, and muscle fatigue.

The prevalence of dysfunction was 33.8 %, in a report with a sample of 32 HIV patients, using FAI as a diagnostic tool.^7^ In another study, the authors observed an increase in the presence of bruxism in 60 HIV patients, according to the duration of antiretroviral therapy.^11^ The prevalence of TMD in the HIV population suggested it may be linked to other predisposing factors like oral conditions^9^ and muscle metabolism.^12^ Psychosocial factors and the social context would also contribute to TMD onset. These changes may be an alert to a complaint that should be underestimated in HIV care. Other associated factors were female sex and being older than 50 years. This is following previous evidence demonstrating a higher frequency of TMD in women and older patients.^1^

Depression was significantly associated with TMD in HIV patients in the current study, after logistic regression (OR = 0.960, 95 % CI 0.922‒1.000). A study with 109 adult patients observed a positive association between depression and joint pain.^6^ In addition, another study showed moderate to severe depression in 95.45 % of the patients who presented TMD.^7^ The association between depression and TMD can be justified by the impact of symptoms of the joint disease on the patient's quality of life.^5^ In addition, depression can be associated with pathological factors and psychosomatic processes of some systemic diseases, making causality uncertain in this context.^21^^,^^22^ It is important to note that depression is a multifactorial disease that involves social, demographic, and health factors, and can also be triggered or exacerbated by pain-related processes, especially chronic pain.^6^

In this study, frailty was not associated with TMD. Despite this finding, the term oral frailty has already been proposed by some authors in the literature that relate a decrease in oral function with aging and exacerbation of systemic frailty.^10^^,^^16^ Chronic pain and changes in chewing are frequent findings in these studies and can lead to TMD or exacerbate a pre-existing condition. Joint pain and muscle function were evaluated in ten participants in a previous study that used a structured questionnaire. Based on the results, the authors proposed the development of an oral and maxillofacial Frailty Index.^10^ Although there is no consensus between studies on TMD and FS, adult HIV patients diagnosed with FS can present early orofacial changes, which reinforces the importance of multidisciplinary attention for the integral treatment of such individuals.

TMD was significantly related to difficulty in opening the mouth (71.4 %), parafunctional habits (67.7 %), joint pain (66.7 %), and muscle fatigue (61.8 %), these symptoms frequency increased with severity of joint involvement. Also, difficulty in opening the mouth, muscle fatigue, and joint noises showed a greater association in individuals with HIV in the logistic regression. In 2002, a prospective study reported bruxism, clicking, and deep bite as predictive signs of long-term dysfunction.^4^ In previous reports, differences between studies about symptoms in TMD may be a consequence of the study's population, the diagnostic tools used, and the degree of involvement when the dysfunction was diagnosed. Signs and symptoms such as clicking, muscle and joint pain should not be ignored, because they have a potentially important predictive value on disease evolution.

### Limitations

This is a cross-sectional study which does not allow us to establish a cause-and-effect relationship or association between the variables studied. Although the study was conducted at an HIV reference center, the sample size we used is larger than most of the available studies on this topic and provides more consistent evidence of the detected associations. Despite these limitations, the study brings a new approach to TMD in patients with HIV. Larger, prospective studies are needed to clarify the frequency and determinants of TMD in the HIV population, especially in those presenting with depression.

## Conclusion

The present work supports the hypothesis of an association between female sex, depression, difficulty in opening the mouth, muscle fatigue and joint noises, and TMD in HIV patients. These findings also reinforce the need for healthcare policies focusing on the stomatognathic system in people living with HIV, especially in women. Symptoms associated with TMD can be limiting and impact the quality of life. Prospective studies assessing the relationship between mental health, gender, and temporomandibular dysfunctions in this population are necessary for a better understanding of this association.

## Conflicts of interest

The authors declare no conflicts of interest.
